# Temozolomide increases the generation of cell heterogeneity in ERK activity in glioma cells

**DOI:** 10.1007/s00109-026-02644-2

**Published:** 2026-01-29

**Authors:** Karine Rech Begnini, Julia Caroline Marcolin, Luiza Cherobini Pereira, Letícia Cunha Pereira de Souza, Frederico Kraemer-Mattos, Daphne Tórgo, Carolina Machemer, Georgia da Silva Goulart, Andrew Oliveira Silva, Jephesson Alex Floriano dos Santos, Guido Lenz

**Affiliations:** 1 Labsinal, Departamento de Biofísica, Universidade Federal Do Rio Grande Do Sul (UFRGS), Rua Bento Gonçalves, 9500, Prédio 43431 – Lab. 115, Porto Alegre, RS 91501-970 Brazil; 2 Centro de Biotecnologia, Universidade Federal Do Rio Grande Do Sul (UFRGS), Porto Alegre, RS Brazil; 3 Laboratório de Células Tecidose Genes,, Centro de Pesquisas Experimentais , Hospital de Clínicas de Porto Alegre, Porto Alegre, RS Brazil; 4 Programa de Pós-Graduação Em Biologia Celular E Molecular, Universidade Federal Do Rio Grande Do Sul, Porto Alegre, RS Brazil

**Keywords:** Phenotypic heterogeneity, Non-genetic heterogeneity, Clonal cells, MAPK signaling heterogeneity, Colonies

## Abstract

**Abstract:**

ERK activity governs diverse cellular responses and has significant implications in cancer biology and treatment. Cellular heterogeneity is a major feature of cancer and a barrier for therapy success, allowing cancer cells to adapt and survive in challenging environments. Here, we used a genetic live-cell reporter to explore the heterogeneity of ERK signaling activity within cellular populations and colonies of glioblastoma (GB) cells. GB cells showed a wide spectrum of ERK activation levels in basal culture conditions and throughout state transitions. Treatment with the chemotherapeutic agent temozolomide increased the phenotypic heterogeneity in ERK activity within cells even in clonal populations. Using the MEK inhibitor trametinib in combination with temozolomide to homogenize ERK activity reduced cell fitness in colonies and decreased fractional killing in GB clonal cells. Our study contributes to the growing understanding of the complexity in ERK activity and dynamics, pointing out the consequences of cell-to-cell ERK phenotypic variability in fitness and therapy survival. The complexity of ERK signaling phenotypes in the context of chemotherapy treatment is shown, offering valuable insights about the intricacies of ERK signaling heterogeneity and chemotherapy treatment.

**Key messages:**

- Heterogeneity in ERK activity in live GBM cells is high in basal culture conditions.
- Temozolomide alters ERK activity in GBM cells and generates phenotypic heterogeneity.
- Clonal populations behave heterogeneously in ERK activity, and this heterogeneity impacts fitness.
- Targeting the generation of ERK heterogeneity reduces fitness and fractional killing in GBM colonies.

**Supplementary Information:**

The online version contains supplementary material available at 10.1007/s00109-026-02644-2.

## Introduction

Cell heterogeneity is a common characteristic of many cancers, usually linked to tumor aggressiveness, therapy resistance, and poor prognosis. Tumor heterogeneity of the genome [1–3], as well as transcriptomic and other non-genetic sources, contributes to producing a diverse array of cell phenotypes within a tumor [4–6]. Genomic heterogeneity contributes to the generation of clonal and sub-clonal populations found in the majority of cancers; however, the selective process of mutations is slow and provides limited flexibility for diversification in a dynamic environment [7]. On the other hand, heterogeneity in cell phenotypes driven by non-genetic sources is dynamic and can shift its features and consequently the phenotype of the cell, over short periods of time [8, 9]. Drivers for this dynamic phenotypic heterogeneity are multifactorial including response to signaling inputs [10, 11], intrinsic cellular states, like the expression of specific mRNAs, regulation of mRNA translation, cell cycle phase and signaling nodes [9, 12–14], and paracrine inputs from the tumor microenvironment [15, 16].

Therapy resistance is a major problem in cancer management. Even within genetically identical cells, the response to therapy is heterogeneous, resulting in fluctuating levels of cell death priming and fractional killing [13]. This, in turn, impacts the capacity of therapeutic agents to eliminate all cells, allowing for the selection of resistant clones that emerge from single cells exhibiting tolerant phenotypes [17]. Untreated cancer cell populations from prostate [18], lung [19], glioblastoma (GB) [20], and melanoma [17] contain drug-tolerant phenotypes that are transient and reversible and occur at low frequency. The cell populations emerging from drug-tolerant single cells are molecularly, morphologically, and functionally distinct, evidencing the capacity of these cells to promote phenotypic tumor cell repopulation [17]. This implies that cancer cells utilize dynamic cell-shifting phenotypes as a survival strategy to shield themselves from lethal conditions, highlighting that the understanding of therapy resistance involves differentiating between static and dynamic cellular states and detecting phenotypes that can shift and only occur at specific time frames [21, 22].

The extracellular signal-regulated kinase (ERK) controls essential cellular processes in response to extracellular inputs [23]. The emergence of live cell reporters to ERK signaling [24, 25] illuminated the complexity and allowed a deeper understanding of the importance of dynamics on ERK response to different stimuli [26], challenging the view of ERK signaling response based mainly on the duration of ERK activity, which was classically separated in short or long [27]. ERK activity dynamics, namely duration and frequency of ERK pulses, is involved in cell fates’ decision both in physiological conditions, like cell cycle decisions and cell migration [28, 29], and tumorigenesis [30–32]. In cancer, it is becoming clear that mutations and other non-genetic disturbances in Ras-Raf-MEK-ERK signaling do not simply hyperactivate the pathway in a homogeneous way in all cells but rather change signaling transmission properties and dynamics [32–34], which can be different in each cell depending on the extracellular inputs a particular cell receives [15] and its current cell state [17].

The ERK pathway is frequently altered in GB through amplification and activating mutations in the epidermal growth factor receptor (EGFR) gene, which are invariably present in a heterogeneous fashion [35]. EGFR-mutated cells increase growth, migration, and infiltrative properties in the EGFRwt cells [35, 36] pointing to the positive interaction of these heterogeneous populations. GB cells that survive to TMZ in a mouse model present cancer stem cell properties and are relatively quiescent [37], with super-enhancer-associated genes predicting ERK pathway inhibitor sensitivity [38]. Cells from the tumor core and from the peritumoral brain express different levels of transcription factors AP-1 and BACH1, which, when deleted, sensitize cells to ERK pathway inhibition [39], indicating that regional heterogeneity is also relevant to the response to therapeutic agents.

Here, we used genetically encoded cells with a fluorescent ERK translocation reporter (ERK-KTR) to monitor ERK activity in live cells. We showed that treatment with temozolomide increases the basal heterogeneity of ERK activity in glioblastoma cells. Phenotype homogenization of ERK activity decreases fractional killing in clonal populations of glioma cells after temozolomide therapy.

## Material and methods

### Plasmids and reagents

Temozolomide (Sigma-Aldrich, catalog number T2577), trametinib (MedChem, catalog number HY-10999/CS-0060), epidermal growth factor human (MedChem, catalog number HY-P7109), PD184352 (Sigma-Aldrich, catalog number PZ0181), DMEM low glucose (Gibco, catalog number 31600034), DMEM high glucose (Gibco, catalog number 1210046), DMEM without phenol red (Gibco, catalog number 11054001), fetal bovine serum (Gibco, catalog number 12657029), penicillin streptomycin (Gibco, catalog number 15140122), MycoAlert™ Plus Buffer (Lonza, catalog number LT27-286), ERK-KTR lentiviral plasmid (pLentiPGK Puro DEST ERKKTRClover, Addgene #90227), Apple-53BP1trunc (Apple-53BP1trunc, Addgene #69531), polybrene (EMD Millipore, catalog number TR1003G), PEI (ethylenediamine branched) (Sigma-Aldrich, catalog number 408719), puromycin (Merck, catalog number P8833).

### Cell lines

In this study, we used A172 glioblastoma cell line (ATCC, catalog number CRL-1620), U-251 MG glioma cell line (STR validated in October 2018 by the Banco de Células do Rio de Janeiro), U138 MG glioblastoma cell line (ATCC HTB-16), and MRC5 lung fibroblast cell line (ATCC, catalog number CCL-171). A172, U-251 MG, and U138 MG glioma cell lines do not carry EGFR amplification or EGFRvIII mutation or other mutations in the MAPK pathway [40]. U-251 MG carries PTEN and TP53 loss-of-function mutations [40]. All cell lines were cultured in Dulbecco’s Modified Eagle’s Medium (DMEM) supplemented with 2 mM L-glutamine, 100 U/ml penicillin, 100 mg/ml streptomycin, and 10% fetal bovine serum. Hek-293 (ATCC, catalog number CRL-1573) cell line was cultured in DMEM High glucose (4.5 g/L) supplemented with 2 mM L-glutamine, 100 U/ml penicillin, 100 mg/ml streptomycin, and 10% fetal bovine serum. Cells were maintained in an incubator with constant temperature (37 °C), CO2 (5%), and humidity. Cell cultures were frequently tested for *Mycoplasma* contamination using MycoAlert kit following manufacturer’s instruction.

A172, U-251 MG, and U138 MG glioma cells and MRC5 lung fibroblast cells stably expressing ERK-KTR fluorescent reporter [25] and/or the nuclear marker Apple-53BP1trunc [41] were generated via lentiviral transduction. Live cell measurement of ERK activity was performed on an IncuCyte S3 or SX1 device (Sartorius). Whenever indicated, cells were stimulated with EGF or treated with temozolomide (TMZ) or TRAM at specific doses. At least 1 h prior to imaging, media were changed to imaging media (DMEM without phenol red with 10% or 0.5% of FBS). Cells were imaged in green and red fluorescence every 10 min for 3 h with imaging starting 30 min prior to EGF stimulation or 3 h prior to TRAM and TMZ treatment (10× magnification). Neither the average of ERK activity nor the phenotypic space changed if using cells from image fields or the whole well (Fig. S1A). For colonies’ growth, plates were imaged every 24 h in red fluorescence. The number of live cells was daily manually determined for fitness and fractional killing quantification. The identity of the colony was determined by its location on the plate using ImageJ software [42].

**Fig. 1:**
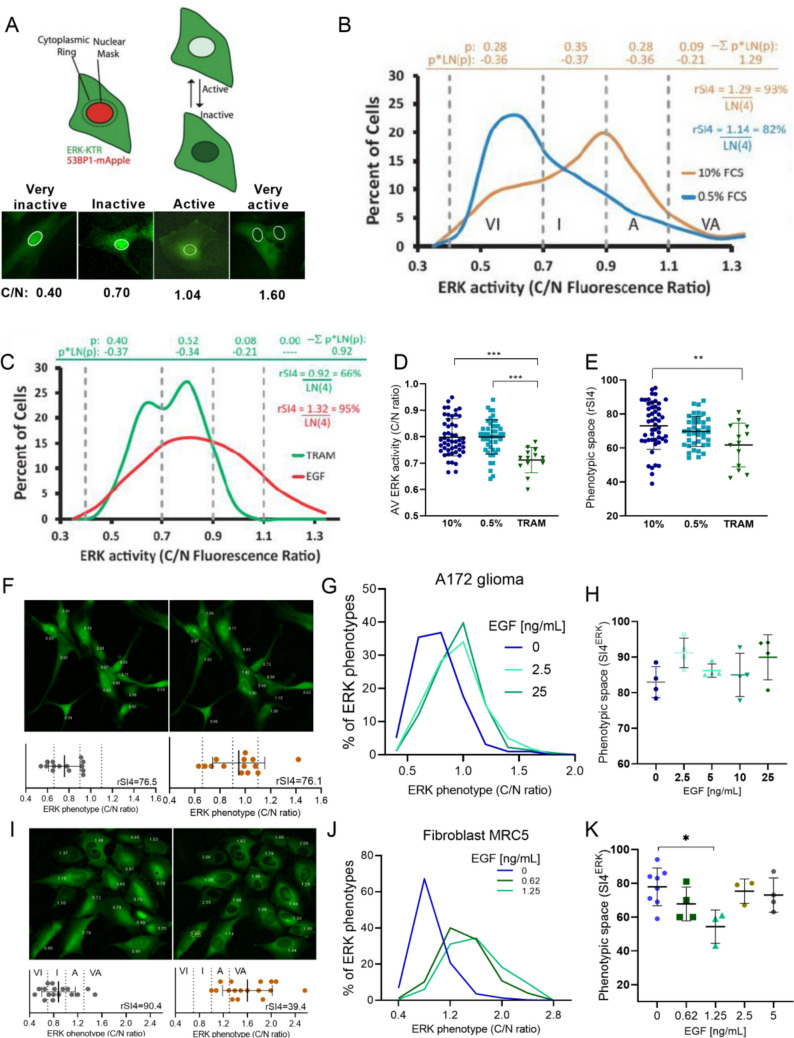
Phenotypic space of ERK activity in glioblastoma cells. **A** ERK activity was measured by the cytoplasmic/nuclear (C/N) ratio of the green fluorescence of cells expressing ERK-KTR and 53BP1-Apple. **B** Four levels of ERK activity were defined as very inactive (VI), inactive (I), active (A), and very active (VA) based on the distributions of ERK activity of A172 cells grown in 10% FBS (orange line) or 0.5% FBS (blue line) for 48 h and **C** 20 nM trametinib for 2 h (green line) or 2.5 ng/ml of EGF for 15 min (red line) after 48 h of serum deprivation. Relative Shannon Index of 4 groups (rSI4 or SI4^ERK^) calculations are indicated. **D** Average ERK activity and **E** phenotypic space (rSI4) occupied by cells treated as in **B** and **C**. Each dot represents an image field with at least 20 cells (10% *n* = 2085 cells; 0.5% *n* = 4466 cells; TRAM *n* = 362 cells). **F** Representative image field of A172 glioma cells and **I** MRC5 fibroblast cells before (left) and after (right) 15 min of 2.5 ng/ml of EGF treatment (20× magnification). The numbers in the image represent the C/N ratio of each cell. The distribution of cells’ phenotypes and SI4^ERK^ for this group of cells is shown in the graphs below the images. **G** Distribution of cells treated with EGF after 48 h of serum deprivation in A172 glioma cells and **J** MRC5 cells. **H** SI4^ERK^ occupied by A172 glioma cells and **K** MRC5 cells after 15 min of EGF treatment. Each dot represents an image field with at least 20 cells (A172 *n* = at least 350 cells per EGF dose; MRC5 *n* = at least 186 cells per EGF dose) One-way ANOVA. EGF, epidermal growth factor; TRAM, trametinib; rSI4 or SI4^ERK^, relative Shannon Index of 4 groups of ERK activity); *p < 0.05; **p < 0.01; ***p < 0.001

A172 and MRC5 image analysis and ERK-KTR fluorescence quantification were determined using forNMA, an in-house pipeline written in Python (version 1.0, unpublished). For each cell, the Apple-53BP1 red fluorescence channel was used for nuclear segmentation, and a cytoplasmic ring was determined by increasing the nuclear area in 3 pixels. Mean fluorescence intensity values on the ERK-KTR green channel for each cell (nuclear and cytoplasmic ring) were extracted and used to calculate ratios, meaning that the ERK signaling activity was quantified through the relative cytoplasmic to nuclear fluorescence (C/N) ratio [25]. U-251 MG image analysis and ERK phenotype classification were conducted through visual assessment of ERK-KTR fluorescence by four independent evaluators. Each cell was categorized as very inactive, inactive, active, or very active based on its nuclear fluorescence (Fig. 1A), and the distribution of cells across these categories was subsequently used to quantify phenotypic heterogeneity using the Shannon Index.

### ERK activity

For ERK activity measurements (average, phenotypes identification and SI4^ERK^), A172_ERK-53bp1, U-251 MG_ERK, and MRC5_ERK-53bp1 cell lines were seeded at a density of 10,000 cells onto a 6-well plate. The next day, media were exchanged to 10% or 0.5% FBS for 48 h prior to stimulation or imaging. Whenever indicated, cells were stimulated with EGF or treated with TRAM at specific doses. At least 1 h prior to imaging, the media were changed to imaging media (DMEM without phenol red with 10% or 0.5% FBS). For the phenotype identification and SI4^ERK^ determination, cells were transferred to an IncuCyte S3or SX1 (Sartorius) and imaged in green and red fluorescence once at the end of specific treatments (TRAM; 10% or 0.5% FBS) (10× magnification). Each treatment condition had at least 6 image fields per well and at least 3 experimental replicates.

For the Intermitotic time (IMT), A172_ERK-53bp1 cells were imaged in green and red fluorescence every 30 min, and imaging tracking of individual cells was performed manually. IMT was calculated in minutes from the birth of a cell to its next division. Average ERK activity of the last 5 h prior to mitosis was considered ERK activity of the mother cell.

For dynamics analysis of average ERK activity and SI4^ERK^, cells were imaged in green and red fluorescence every 10 min for 3 h with imaging starting 30 min prior to EGF stimulation or 3 h prior to TMZ treatment (10× magnification). Each treatment condition had at least 6 image fields per well. EGF stimulation had 1 experimental replicate, and the TMZ treatment condition had 3 experimental replicates.

### Population doubling (PD)

A172_ERK-53bp1 cells were plated onto 24-well plates at a density of 100,000 cells. Plates were kept on an IncuCyte device and imaged every 24 h in red fluorescence (4× magnification). Untreated wells were passaged every 3 days to keep the exponential growth of cells and each time the same number of cells was reseeded. The number of live cells was daily manually counted for PD determination as follows:

$$PD=({\mathrm{log}}_{2}\left(fN\right)-{\mathrm{log}}_{2}\left(iN\right))$$

where *fN* is the number of cells in the well at the day of image, and *iN* is the number of cells in the well at the day before [36]. The cumulative values of PD (CPD = sum of all PDs) were plotted versus days of culture.

### Colonies’ experimental conditions

For colonies’ growth, a mix of A172 and A172_ERK-53bp1 cell lines was plated at a density of 200 cells (proportion of 198:2) in a 96-well plate. Plates were kept on an IncuCyte device and imaged every 24 h in red fluorescence (4× magnification). Single cells expressing the nuclear marker fluorescence were identified on day 1, and their growth to colonies was manually tracked for 14 days using images. The number of live cells was daily manually determined for fitness and fractional killing quantification, and the identity of the colony was determined by its location on the plate. TMZ (100 µM for 3 h), TRAM (20 nM for 24 h), or a combination of TMZ (100 µM for 3 h) and TRAM (20 nM for 24 h) treatment was added on day 4 of growth for treated colonies. Colonies that did not reach a minimum growth of 4 cells at day 4 were excluded from analysis. For ERK activity (average and SI4^ERK^) measurement, colonies were imaged in green and red fluorescence every 10 min for 3 h on day 4 (before treatment addition) and day 7 of growth (3 days after treatment removal) (10× magnification).

Fitness of colonies was determined by total cell number at a specific time point, colony size (log_2_ of cell number), or expressed as cumulative population doubling (CPD) [35]. For colony size and cumulative population doubling analysis, the value of 0.5 was used to denote the total elimination of cells to avoid undefined log values. The growth rate of colonies after treatment was calculated as:

$$GR={\mathrm{log}}_{2}\left(CSa\right)-{\mathrm{log}}_{2}\left(CSb\right)/days$$

where CSa is the colony size after treatment, and CSb is the colony size before treatment [13].

Fractional killing of colonies after treatment was determined as inversely proportional to lethal fraction (LF) [38]. LF was calculated as LF = 1 − (*N*_alive cells_/(*N*_dead cells_ + *N*_alive cells_). The number of live cells in each colony was daily counted manually and used to infer the occurrence of death among cells (every time a colony had fewer alive cells than previously, the missing cells were considered dead).

### Shannon Index calculation

The Shannon’s diversity index (H or SI) was used to quantify the distribution of cells across ERK activity levels and the distribution of colonies across lethal fraction (LF) induction groups. SI is calculated as:

$$H(SI_{{\mathrm{s}}} ) = - \sum\limits_{i = 1}^{s} {(p_{{\mathrm{i}}} } \log_{{\mathrm{s}}} p_{{_{{\mathrm{i}}} }} ) \times 100$$

where *p*_i_ is the proportion of cells in each ERK level/LF groups, and *S* is the number of groups. SI ranges from 0 to 100, implying that a value of 0 means that all cells are in the same group while a value of 100 means that cells are equally distributed across all groups.

## Results

### Heterogeneity in state transitions in ERK activity

Given the importance of ERK activity in cancer, we used cells expressing a kinase translocation reporter (ERK-KTR) to describe the array of ERK activity phenotypes in live glioblastoma cells, herein called ERK phenotypic space. KTR fluorescence serves as a proxy for kinase activity because the reporter translocates from the nucleus to the cytoplasm in response to ERK-dependent phosphorylation. The KTR detects the ERK activity based on the relative cytoplasmatic to nuclei (C/N) fluorescence intensity of the reporter [25] (Fig. 1A). To describe the occupancy of ERK phenotypic space, we used the distributions of ERK activity of cells grown in media containing 10% or 0.5% fetal bovine serum (FBS) (Fig. 1B) or treated with the MEK inhibitor trametinib (TRAM) or epidermal growth factor (EGF) (Fig. 1C). These distributions were used to define four levels of ERK activity, namely very inactive (VI), inactive (I), active (A), and very active (VA) (Fig. 1A-C). The proportions of cells in these four levels were used to calculate the Shannon diversity Index (SI), which reflects the degree of occupancy of the phenotypic space by a population of cells and its phenotypic heterogeneity. The SI of these 4 groups for ERK activity (SI4^ERK^) ranged from 66% for TRAM treated cells, in which most cells were in the very inactive and inactive states with no cell in the very active state, to 95% in the EGF treated group, in which the cell population was more evenly distributed among the four levels, indicating a more complete occupancy of the phenotypic space (calculations in Fig. 1B and C). Using other numbers of ERK activity groups produced similar SI^ERK^ values to 4 groups, indicating that the number of groups and phenotype thresholds does not greatly affect the measured heterogeneity (Fig. S1B).

Average and phenotypic space of ERK activity in a population of cells are not necessarily linked; however, treatments that approximate the average to the extremes tend to reduce heterogeneity. Inhibition of MEK with trametinib reduced the average ERK activity and the SI4^ERK^ (Fig. 1D and E). EGF increased the average ERK activity (Fig. S1C and S1 E) and slightly increased SI4^ERK^ in glioma cells (Fig. 1F, G, and H). Surprisingly, a fibroblast cell line had a homogeneous increase in ERK activity upon EGF stimulation even with low doses thus producing a reduction in SI4^ERK^ (Figs. 1I, J, and K and S1D). While in both cell lines EGF increased average ERK activity, only in the glioma cell line is this produced without a decrease in the phenotypic heterogeneity.

### Clonal populations are heterogeneous in ERK activity

**Fig. 2:**
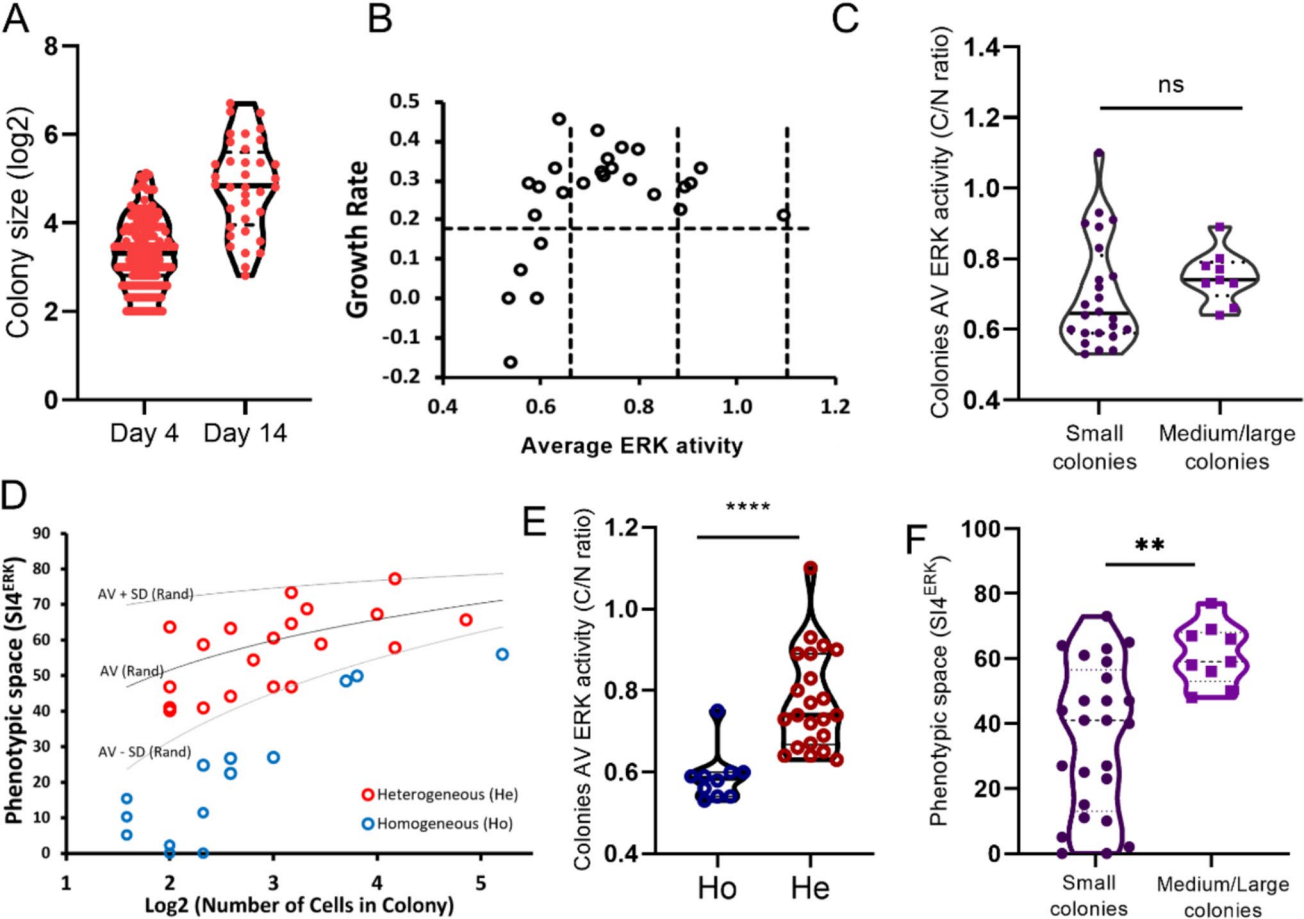
Fitness heterogeneity and ERK phenotype heterogeneity in clonal cells. **A** Fitness quantification (log_2_ colony size) of untreated colonies on day 4 (*n* = 122) and day 14 (*n* = 36) after plating. **B** Colonies were categorized according to ERK activity status (day 4) in relation to growth rate between day 4 and 14. **C** Average ERK activity of small (up to 10 cells) and medium/large (10–30 cells or > 30 cells) colonies at day 4. Each dot represents the mean ERK activity of cells of a colony (10× magnification) (*n* = 34 colonies); unpaired *t*-test. **D** Relation of colony size and ERK phenotypic space occupied by colonies. Based on the phenotypic space occupied by randomly assembled populations (average and standard deviation values—gray lines), we categorized the colonies as homogeneous and heterogeneous (*n* = 34 colonies). **E** Average ERK activity for homogeneous and heterogeneous colonies on day 4. Each dot represents the average ERK activity of the cells from a colony imaged every 10 min for 3 h (10× magnification). Unpaired *t*-test. **F** Phenotypic space occupied by small (up to 10 cells) and medium/large (10–30 cells or > 30 cells) colonies at day 4 of growing. Each dot represents the SI4^ERK^ of cells of a colony imaged every 10 min for 3 h (10× magnification) (*n* = 34 colonies). Unpaired *t*-test

To understand how heterogeneous clonal cells are in terms of ERK activity and fitness, we grew colonies and tracked their growth rate and the occupancy of ERK activity phenotypic space (Fig. S2A). Colonies of glioblastoma cells grow in a heterogeneous way, with some growing fast (5 generations in 4 days) and others doubling their population only once in the same timeframe (Fig. 2A). The difference in colony growth rate was maintained during the analyzed period (Fig. 2A) and even on day 14 after plating, 8% of colonies remained with less than 10 cells (Fig. S2B). Colonies exhibited diverse average ERK activity levels, from very inactive (C/N < 0.66) to active (C/N > 0.9) levels (Figs. 2B and S2C). The average ERK activity on day 4 after plating was similar for small (< 10 cells) and medium/large colonies (Fig. 2C), but only very inactive colonies had low growth rates (Fig. 2B and C).

Colonies showed high heterogeneity in ERK activity, with SI4^ERK^ within cells from a colony ranging from 0 to 77% (Fig. 2D). Based on average and standard deviation values of randomly assembled populations (gray lines), we classified colonies as either homogeneous or heterogeneous (Fig. 2D). About 41% of colonies exhibited homogeneous ERK activity, while 59% were as heterogeneous as randomly assembled cell groups (Fig. 2D). Homogeneous colonies usually had a maximum of 8 (2^3^) cells, indicating that the memory of ERK activity of the founder cells lasts at most 3 generations, corroborating our previous data [13, 43]. Heterogeneous colonies have higher average ERK activity among the clonal cells than homogeneous ones (Fig. 2E). Medium (10–30 cells) and large colonies (> 30 cells) are always heterogeneous in ERK phenotypic occupancy while small colonies (< 10 cells) have a broader distribution in the phenotypic spectrum with highly homogeneous and heterogeneous colonies (Fig. 2F).

### TMZ treatment increases ERK activity and heterogeneity in glioblastoma cells and colonies

**Fig. 3:**
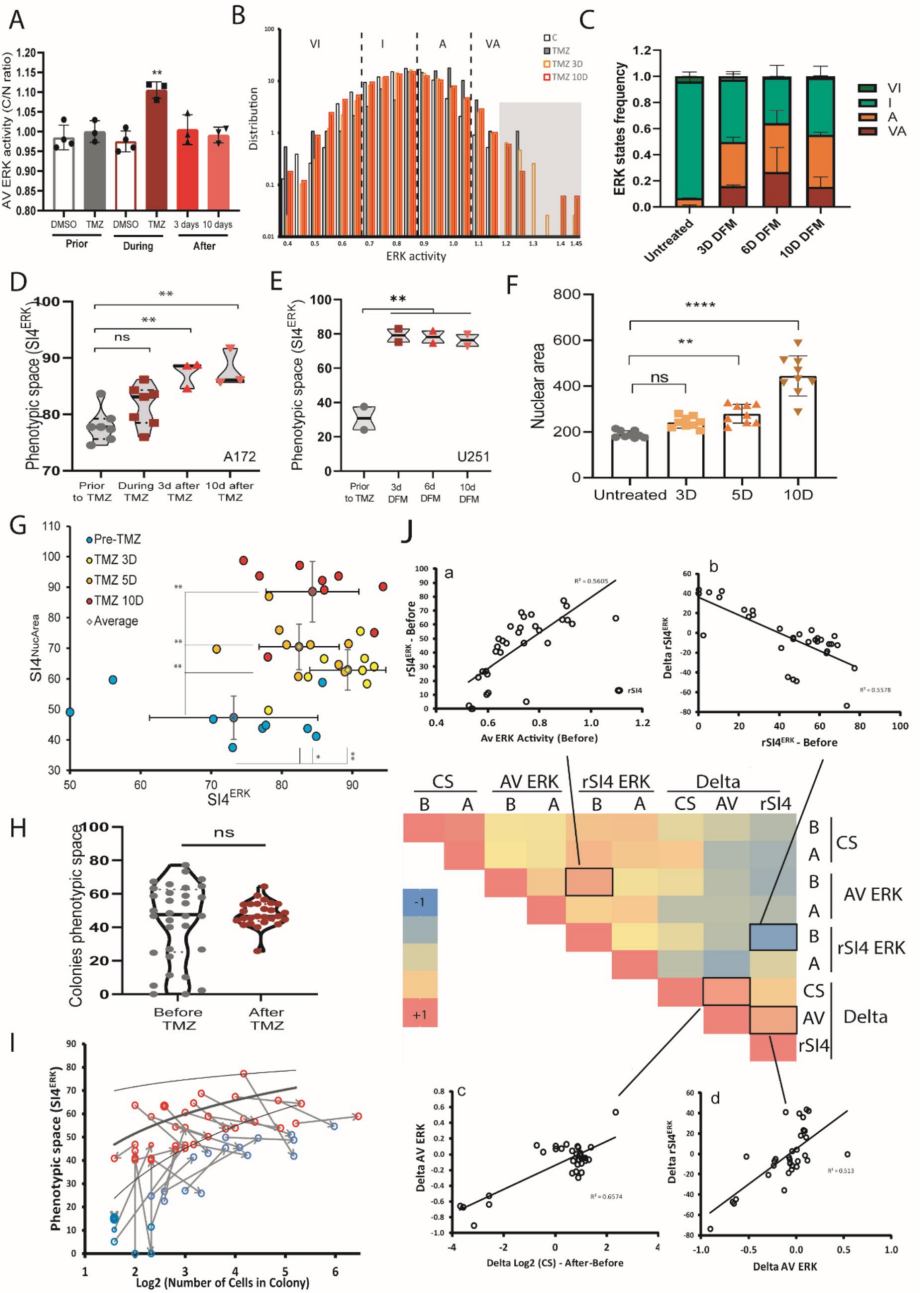
Impact of TMZ on ERK phenotype heterogeneity. **A** Average and **B** distribution of ERK activity of A172 cells treated with TMZ (100 mM for 3 h). Cells were imaged every 10 min for 12 h prior to TMZ treatment, during 3 h of treatment, and for 12 h 3 or 10 days after treatment withdrawal. Each dot represents the time-averaged ERK activity of an image field with at least 20 cells (prior to TMZ *n* = 1834 reads, during TMZ *n* = 268 reads, 3 days after *n* = 9683 reads, 10 days after *n* = 3671 reads). DMSO was used in the same volume as TMZ for control of mechanical ERK stimulation. One-way ANOVA. **C** Distribution of ERK states frequency prior to TMZ treatment, and 3, 6, or 10 days after treatment withdrawal in U-251 MG cells. **D** Phenotypic space occupied by A172 or **E** U-251 MG cells treated as in **A**. One-way ANOVA. **F** Average nuclear area of untreated and TMZ treated A172 cells 3, 5, and 10 days after drug removal. Each dot represents the average of a field with at least 30 cells (10× magnification). One-way ANOVA. **p < 0.005, ****p < 0.0001. **G** Relation of phenotypic space of ERK activity (SI4^ERK^) and nuclear area heterogeneity (SI4^NucArea^) of A172 glioma cells before TMZ treatment and 3, 5, and 10 days after treatment withdrawal. Each dot represents the SI4^ERK^/SI4^NucArea^ of a field with at least 30 cells (10× magnification). Unpaired *t*-test. **H** Phenotypic space of ERK activity occupied by the cells of A172 colonies immediately before (day 4) and 3 days after treatment withdrawal (day 7). Each dot represents the SI4^ERK^ of a colony of cells imaged every 10 min for 3 h (10× magnification) (untreated *n* = 32; TMZ treated *n* = 26). Unpaired *t*-test. **I** Change in SI4^ERK^ of each colony after TMZ treatment and its relationship with colony growth. Each dot represents the SI4^ERK^ of a colony of cells imaged every 10 min for 3 h (10× magnification). Arrows indicate the pathway from initial to final SI4^ERK^ and colony size of each colony. Blue dots indicate homogeneous colonies, and red dots indicate heterogeneous colonies. **J** Correlations among colony size (CS), average ERK activity of the colony (AV ERK), and phenotypic space of ERK activity (SI4^ERK^), together with the deltas (∆CS, ∆AV^ERK^, and ∆SI4^ERK^) of these features from day 4 (B, before TMZ) and day 7 (A, 3 days after TMZ withdrawal). Relevant positive and negative correlations are shown in detail (**J** (a, b, c, d))

Since chemotherapy survival represents a huge challenge for cancer cells, we aimed to evaluate the impact of TMZ, the standard treatment for gliomas, on the phenotypic heterogeneity of GB cells. TMZ, at concentrations within the range of peak plasma levels observed in patients undergoing therapy [43], increases average ERK activity in glioma cells during treatment (Fig. 3A, B, and C). Heterogeneity in ERK activity increases only after 3 days of TMZ treatment and persists for at least 10 days following TMZ withdrawal (Fig. 3D and E), mainly due to the increase in the proportion of active and very active cells (Fig. 3B (shadowed area) and C), despite a reduction of the average ERK activity to pre-treatment levels (Fig. 3A). Average ERK activity and SI4^ERK^ of imaged fields did not correlate in untreated and TMZ-treated conditions (Fig. S3A). Example of the dynamics in ERK activity and SI4^ERK^ of a field of at least 50 cells indicated an increase in average activity during TMZ addition followed by an increase in SI4^ERK^ 72 h later, despite reduction in average activity to pre-treatment levels (Fig. S3B). As ERK signaling regulates cell proliferation and senescence induction and maintenance [23, 44], we evaluated nuclear area as an indicator of cell cycle phase and on the progression towards senescence [6]. Following TMZ treatment, the average nuclear area increased as most cells arrested in G2 phase of the cell cycle, and some entered senescence (Figs. 3F and S3C). TMZ treatment induced increase both in the heterogeneity of ERK and in nuclear area (SI4^NucArea^) after 3, 5, and 10 days (Fig. 3G). The increase in SI4^NucArea^ also occurs in other GB cell lines (Fig. S3C), but this increase is transitory for U251 and U138 cell lines, despite different sensitivity to TMZ. A similar increase in nuclear area heterogeneity is observed in clonal U251 glioma cells, in which SI4^NucArea^ plateaued around 50% in untreated colonies (Fig. S3I, left) while raising to 90% in colonies treated with TMZ (Fig. S3I, right), indicating the effect of TMZ in the heterogeneity in nuclear area related to phenotypes such as cell cycle distribution and senescence induction.

In clonal populations, TMZ treatment did not change average ERK activity (Fig. S3D) and very inactive colonies maintained low growth rates after treatment (Fig. S3E). On the other hand, TMZ treatment changed the ERK phenotypic space occupied by the cells of colonies after treatment (Fig. 3H and I). We found a reduction in the proportion of colonies with cells that behaved homogeneously (SI4 ^ERK^ < 40) and a reduction of highly heterogeneous colonies (SI4 ^ERK^ > 60), entailing in a uniformity of ERK activity phenotypes occupancy around the median in TMZ-treated colonies (Fig. 3 H and I). For instance, untreated colonies have more similar proportions of colonies among homogeneous, heterogeneous, and highly heterogeneous SI4^ERK^ groups (Fig. S3G upper) than TMZ-treated colonies (Fig. S3G lower). After treatment, only 7% of colonies behaved homogeneously, 4% behaved very heterogeneously, and 89% of colonies were heterogeneous (Fig. S3G).

To understand how ERK activity of every colony was impacted by TMZ treatment, we analyzed the deltas (∆) of colony size, average ERK activity of the colonies, and phenotypic space of ERK activity from day 4 (before TMZ treatment) and day 7 (3 days after TMZ treatment withdrawal) (Fig. 3J). Surprisingly, the occupation of phenotypic space correlated with average activity in colonies (Fig. 3J(a)), and the ones with larger average ERK activity increase (∆AV^ERK^) presented a larger increase in phenotypic space occupancy (∆SI4 ^ERK^) (Fig. 3J (d)) and grew more (Fig. 3J(c)). Corroborating the link of ERK activity and growth rate, cells with very high ERK activity produced daughter cells with faster intermitotic time than cells in the high ERK activity range (Fig. S3F). The ∆SI4^ERK^ was variable among colonies, with some having large phenotypic space modification and others with little or no phenotypic space change, but in general, colonies that started highly heterogeneous decreased their heterogeneity, while highly homogeneous ones increased their heterogeneity (Fig. 3I and I (b)).

### Homogenization of ERK phenotype reduces fitness and decreases fractional killing in glioblastoma colonies

**Fig. 4:**
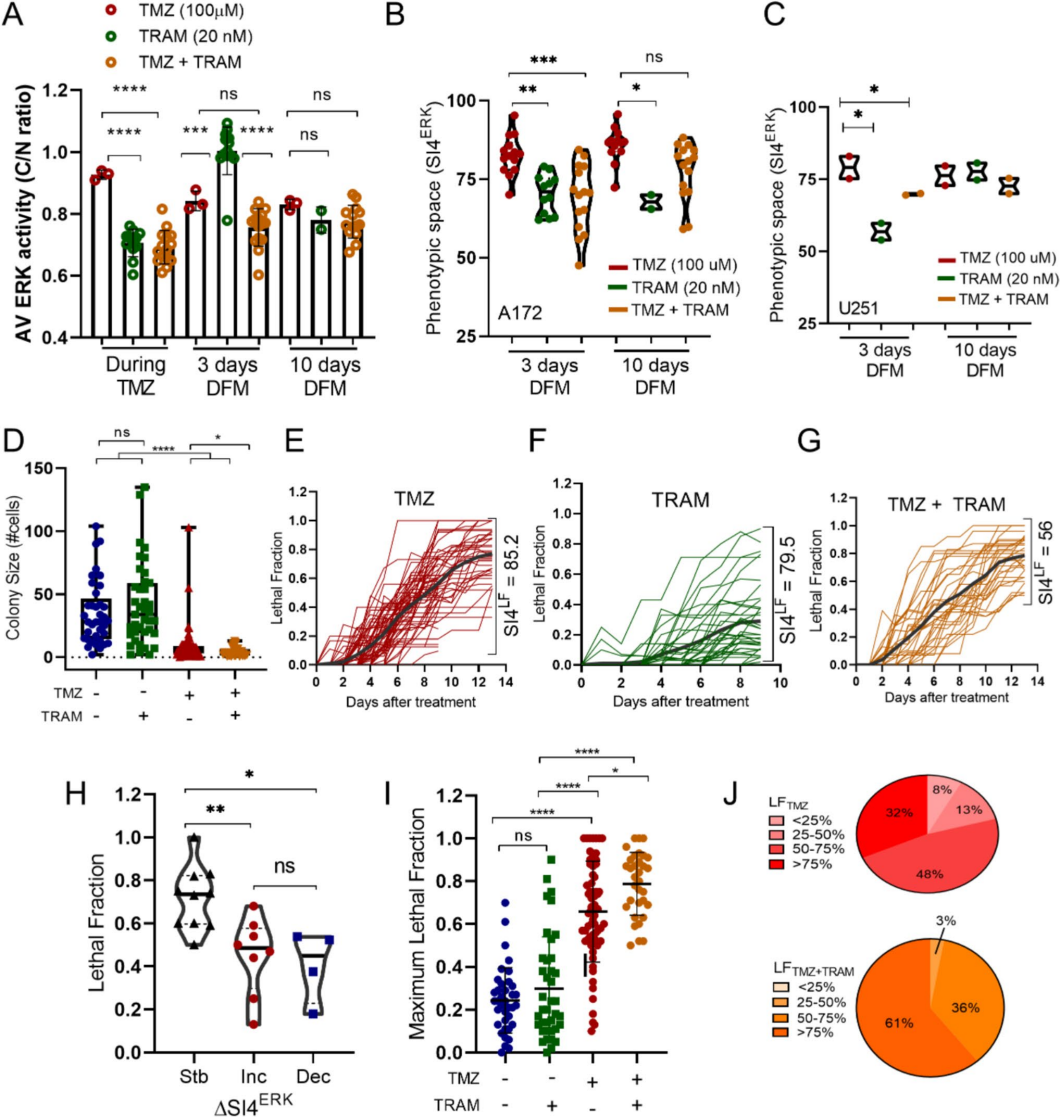
Reduction of ERK phenotype heterogeneity reduces fractional killing of clonal populations. **A** Average of ERK activity from cells treated with TMZ (100 mM for 3 h); TRAM (20 nM for 24 h) or a combination of TMZ and TRAM. Cells were imaged every 10 min for 12 h prior to TMZ treatment, during 3 h of treatment and for 12 h 3 or 10 days after treatment withdrawal. Each dot represents the time-averaged ERK activity of an image field with at least 20 cells (TMZ during *n* = 268, TMZ 3 d *n* = 9683, TMZ 10 d *n* = 3671; TRAM during *n* = 362; TRAM 3 d *n *= 331; TRAM 10 d *n* = 245; TMZ + TRAM during *n* = 428, TMZ + TRAM 3 d *n* = 650; TMZ + TRAM 10 d *n* = 599). One-way ANOVA. **B** Impact of MEK inhibition with TRAM on the SI4^ERK^ 3 or 10 days after drug withdrawal in A172 and **C** U-251 MG cells. Cells were treated as in **A**. One-way ANOVA. **D** Colony size of A172 cells for each treatment type on day 14. Each dot represents a colony. Unpaired, two-sided Mann–Whitney *U* test. **E** Lethal fraction (LF) over time of A172 colonies after TMZ, **F** TRAM or **G** TMZ and TRAM treatments. Heterogeneity in LF induction was calculated as the Shannon Index diversity (SI4^LF^) for 4 equal categories. Black lines represent the average of LF of all colonies. **H** LF of colonies whose phenotypic heterogeneity remained stable (stb), increased (inc) or decreased (dec) 3 days after TMZ treatment. ∆SI4^ERK^ was calculated as SI4^ERK^ after TMZ treatment minus SI4^ERK^ before treatment. ∆SI4^ERK^ was considered stable when it changed to less than 10%. One-way ANOVA. **I** Maximum LF observed after TMZ, TRAM, or TMZ and TRAM treatment. Each dot represents a colony (untreated *n* = 37; TMZ *n* = 64; TRAM *n* = 39; TMZ and TRAM *n* = 33). **J** Proportion of colonies with > 75%, 25–75% or < 25% of death rate at the end of the experiment for TMZ (above) and TMZ and TRAM treatments (lower). LF (lethal fraction); TMZ (temozolomide); TRAM (trametinib); ns, non-significative; *p < 0.05; **p < 0.01; ***p < 0.005; ****p < 0.001

Cells in a colony gradually become different in several phenotypes, including therapeutic tolerance, and drug combinations can be a rationale for specific phenotype homogenizing in cancer cells. To try to homogenize the ERK activity phenotype after TMZ treatment, we used the MEK inhibitor trametinib at a non-cytotoxic dose. The combination of TMZ and TRAM reduced average ERK activity during treatment (Fig. 4A) and decreased heterogeneity induced by TMZ after 3, but not 10 days of drug withdrawal (Fig. 4B and C). This transient phenotype homogenization did not change the effect of TMZ on population cell growth in vitro (Fig. S4A and S4B).

In colonies, TMZ treatment (100 mM for 3 h) strongly reduced the proportion of medium and large colonies of glioma cells while TRAM treatment (20 nM for 24 h) did not impact fitness of colonies (Fig. S4C). The combination of TMZ and TRAM led to a complete elimination of large colonies (Fig. S4C) and a significant reduction in the final colony size (Fig. 4D), even though the colony sizes were similar in all groups on the day when treatment was added (Fig. S4E).

Heterogeneity in therapeutic tolerance can be investigated through lethal fraction (LF), a measure of fractional killing [38], using the number of live and dead cells over time to determine the death rate in a colony [45] (Fig. S4D). TMZ treatment produced a mean LF of 0.77 and killed all cells of 11% of colonies (LF = 1) (Fig. 4E). TRAM treatment produced a mean LF of 0.29 with no colony fully eliminated (Fig. 4F). The LF rates did not correlate with colony size at the moment of treatment addition (Fig. S4F). Both TMZ and TRAM treatments induced heterogeneous LF among colonies (Fig. 4E and F). To understand this variability in cell death induction, we quantified the heterogeneity of LF (SI^LF^). Surprisingly, TMZ treatment (SI^LF^ = 85.2%) and TRAM treatment (SI^LF^ = 79.5%) had similar SI4^LF^ values despite a very different LF mean (Fig. 4E and F).

When ERK phenotype was considered, we interestingly observed that the colonies whose heterogeneity in ERK activity changed less after TMZ treatment (∆SI4^ERK^ changed less than 10%) had higher lethal fraction rates (Fig. 4H), pointing out that the shifting of ERK activity might be a survival strategy. Treating the colonies with the homogenizing ERK activity drug combination, we observed an increased proportion of colonies with higher LF (Fig. 4G and I) and a reduction in LF heterogeneity (SI4^LF^ = 56%) (Fig. 4G). For combined treatment, most colonies showed a LF higher than 0.5, although the mean LF (0.78) was similar to those observed for TMZ alone (Fig. 4E and G). After TMZ treatment alone, 32% of colonies presented a LF higher than 0.75 and 21% had less than 0.5 of LF (Fig. 4J above). When TRAM was combined with TMZ, only 3% of colonies showed LF lower than 0.5, 61% had LF rates above 0.75, and 9% of colonies were fully eliminated (Fig. 4I and J below). Taken together, these data support the notion that TMZ modulates the heterogeneity in ERK signaling phenotypes and that its inhibition can increase the proportion of colonies in which most cells remain sensitive to TMZ.

## Discussion

Phenotypic heterogeneity allows cancer cells to adapt and survive in dynamic environments. The capacity of generating heterogeneity through phenotypic shifting can produce the diversity of phenotypes required to tolerate all the physical, chemical, and biological challenges faced by cancer cells in adverse environments [9, 46]. Our analysis showed that populations of glioblastoma cells maintained in regular culture conditions have high phenotypic space occupancy regarding ERK activity. When challenged with ERK activity inductors or inhibitors, glioblastoma cells remained heterogeneous despite a fluctuation in average ERK activity. Since cellular states have a strong effect on the response of signaling nodes to growth factors and inhibitors and can predict the response of cells in their presence [14], the broader occupancy of ERK phenotypic space of cancer cells might reflect the heterogeneity of cellular states in the glioblastoma population.

Among clonal populations, individual cells respond differently to signals and stress, often due to non-genetic differences associated with transcriptional [9, 47] and signaling states [14, 16]. Those differences result in impacts on the phenome of cells, especially regarding fitness and drug response in cancer [21]. Here, we show that colonies of clonal cells have a broader distribution in the ERK activity phenotypic spectrum, with highly homogeneous and heterogeneous colonies. In line with what we have already shown in our previous works [13, 43], some memory of ERK activity is retained in small colonies, although larger colonies are usually as heterogeneous as randomly assembled cells.

Treatment with TMZ produced a long-term increase in ERK activity phenotypic space occupancy, which remained for at least 10 days, indicating a possible adaptation to the challenging environment of drug exposure. The treatment of clonal populations with TMZ also changed the phenotypic heterogeneity of ERK activity. Colonies of cells that have higher ERK activity before treatment also have a larger increase in phenotypic space occupancy after chemotherapy treatment and left more descendants. This change in heterogeneity in signaling networks can enable specific cellular decision-making since the RAS-ERK signaling network amplifies environmental input fluctuations and paracrine signaling to generate dynamically heterogeneous gene expression states [16] that ultimately define cell fate [14]. We show here an increase in nuclear area heterogeneity following TMZ treatment, which indicates the concomitant presence of cycling cells with cells that present increased nuclear area, an indication of senescence. Although ERK activity represents only one aspect of these complex phenotypes, the link of ERK activity with cell cycle progression may partly explain the heterogeneity observed in nuclear area.

Despite the frequent dysregulation of the MAPK/ERK signaling pathway in gliomas [35], the treatment with MEK inhibitors, such as trametinib, has shown limited efficacy [48]. Even in combination therapies, their effectiveness is often hindered by both intrinsic and acquired resistance mechanisms within tumor cells [49–51] or driven by dynamic cell shifting phenotypes [21, 22]. Here we show that GB cells have a wide spectrum of ERK activation levels, which may partly explain the limited efficacy of MEK inhibitors in GB treatment. When we used trametinib as a pharmacological strategy to reduce the change in ERK phenotypic space in clonal cells, we observed a reduction in fitness after treatments and, more importantly, a decrease in fractional killing of glioblastoma clonal populations. This is in line with other data showing that clonal cells with heterogeneous transcription dynamics are able to survive longer when challenged with different chemotherapeutic drugs [47]. Focusing on heterogeneity, particularly on the capacity of chemotherapeutic agents to generate diversity in phenotypes linked to cancer resistance and growth, is a path forward to better understand the complex response of cancer cells to therapy. This approach helps in designing therapy modulations aimed at reducing heterogeneity, thereby decreasing the likelihood of cells to adopt therapy-tolerant phenotypic states that allow tumor repopulation.

## Supplementary Information

Below is the link to the electronic supplementary material.

ESM 1 (DOCX 1.34 MB)

## Data Availability

The data underlying this article will be shared on reasonable request to the corresponding author.
