# Supplementary material for: Temozolomide increases the generation of cell heterogeneity in ERK activity in glioma cells
Source: J Mol Med (Berl). 2026 Jan 29;104(1):35. doi: 10.1007/s00109-026-02644-2 (PMC12855392; doi:10.1007/s00109-026-02644-2)
Supplement: Supplementary file 1 — (DOCX 1.34 MB) [file 109_2026_2644_MOESM1_ESM.docx]

**Supplementary Figures Legends**

**
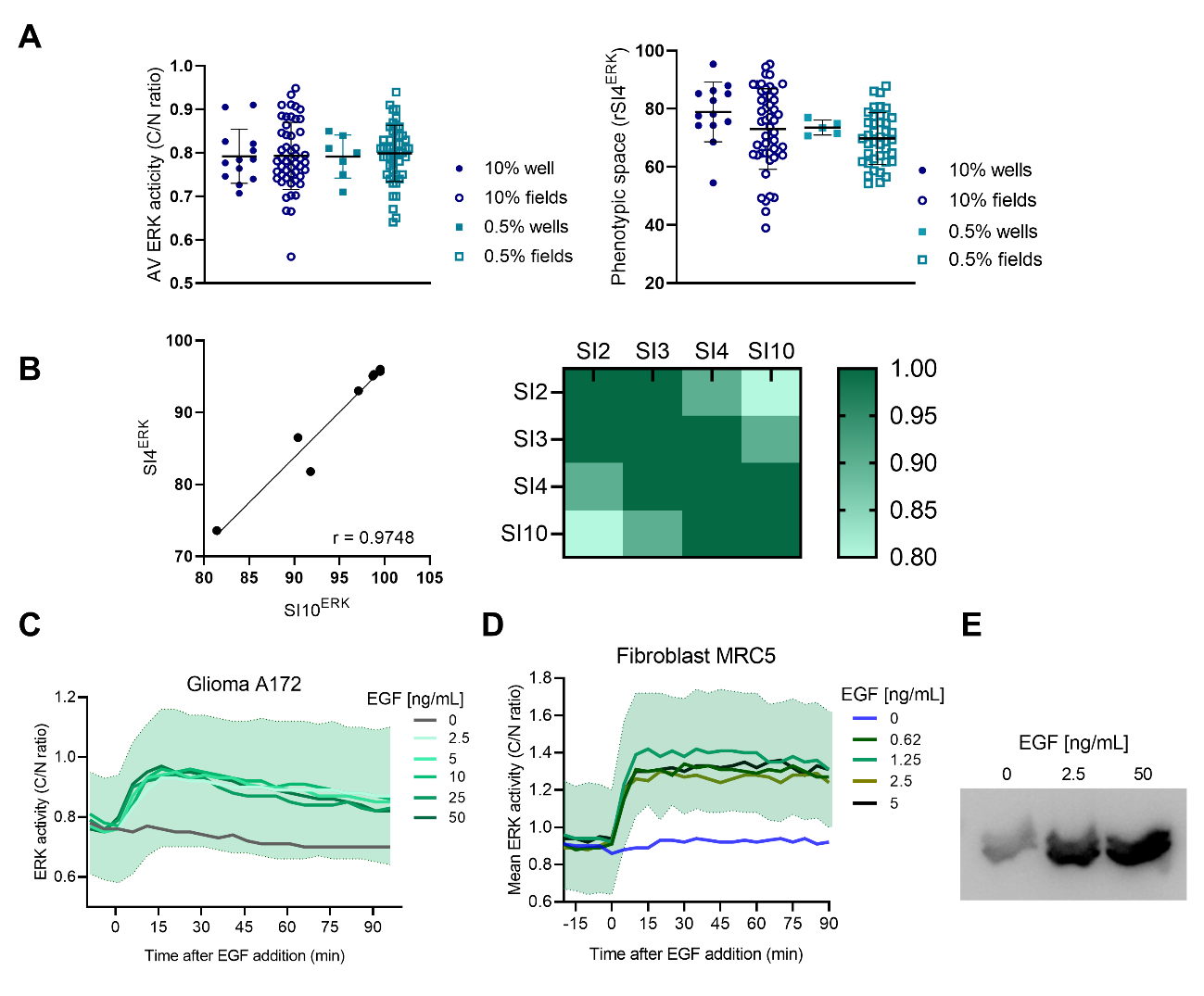
**

**Fig. S1** (A) Comparison of average ERK activity (left) and SI4^ERK^ (right) in A172 cells grown in 10% FBS or 0.5% FBS for 48h when using imaged wells or imaged fields for analysis. Each well represents the average of at least 4 image fields and each field represents at least 20 cells. (B) Comparison between SI calculated using four (SI4) or ten (SI10) ERK phenotypic groups and map of correlation among the SI obtained if using 2, 3, 4 or 10 phenotypic groups equally distributed. (C) Average ERK activity of at least 350 A172 glioma cells after treatment with 0, 2.5, 5, 10, 25 and 50 ng/mL of EGF. Shade area represents standard deviation (SD) of cells treated with 2.5 ng/mL of EGF. (D) Average ERK activity of at least 150 MRC5 fibroblast cells after treatment with 0, 0.62, 1.25, 2.5 and 5 ng/mL of EGF. Shaded area represents SD of cells treated with 1.25 ng/mL of EGF. (E) Western blotting analysis of p44-MAPK and p42-MAPK performed with whole cell extract (40 µg of lysate) of A172 cells starved with 0.5% of FBS for 48h or treated with 2.5 and 50 ng/mL of EGF for 15 minutes.

**
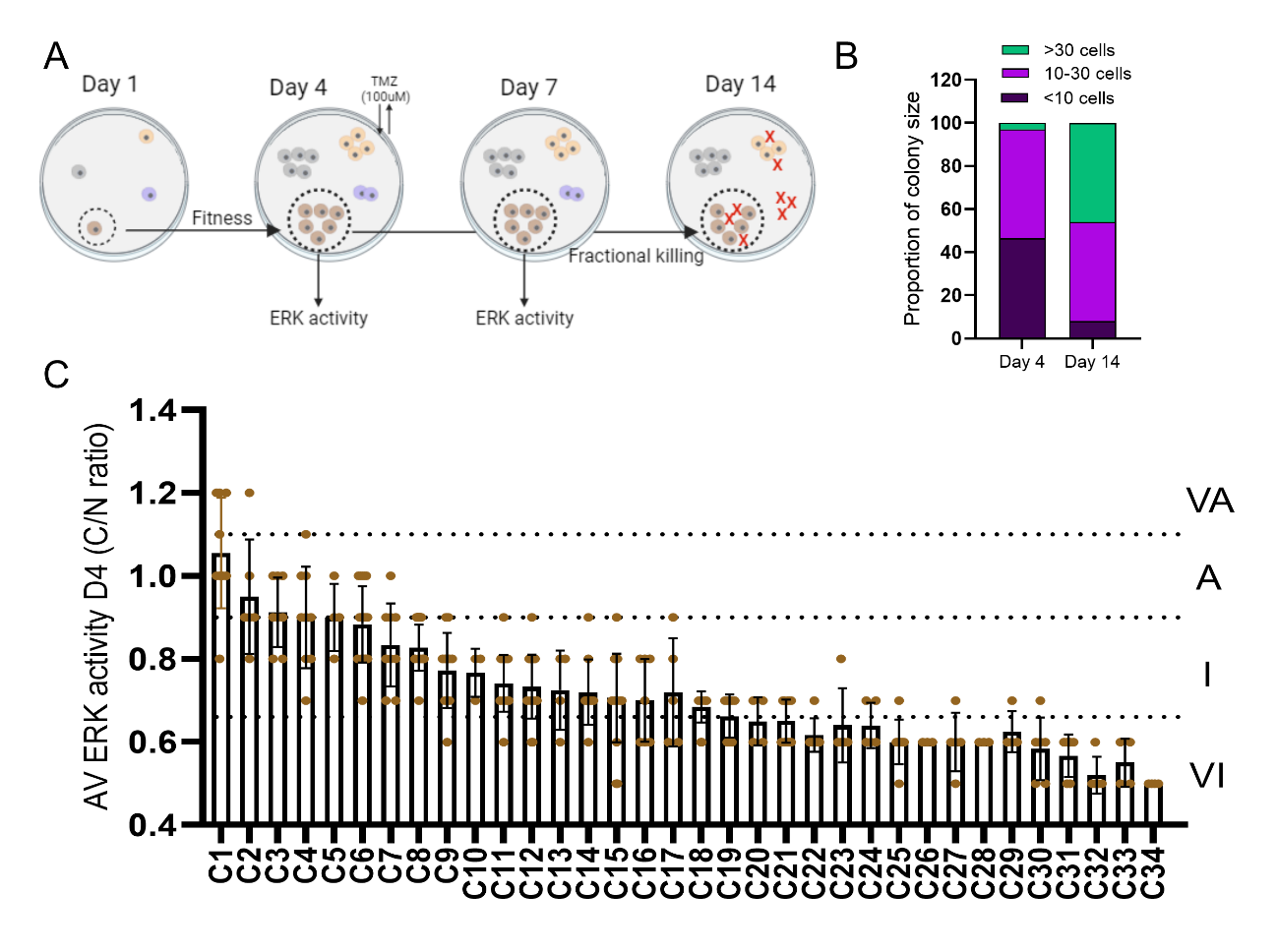
**

**Fig. S2** (A) Schematic of growth analysis of colonies. Unique cells were identified on day 1 and their growth to colonies was manually tracked for 14 days using the nuclear marker fluorescence (Apple-53bp1). TMZ, TRAM or a combination of TMZ and TMZ treatment was added on day 4 of treated colonies. ERK activity (average and SI4^ERK^) was measured on day 4 (before treatment addition) and day 7 of growing (3 days after treatment removal). The number of live cells was daily manually determined to fractional killing quantification. (B) Proportion of colony sizes at day 4 and day 14 of growth (day 4 n=172 colonies; day 14 n=37 colonies). (C) Average ERK activity of colonies (C1-C34) and their position as very inactive (VI), inactive (I), active (A) or very active (VA) state. Each dot represents the average of ERK activity (C/N ratio) of a cell from the colony imaged every 10 min for 3h.

**
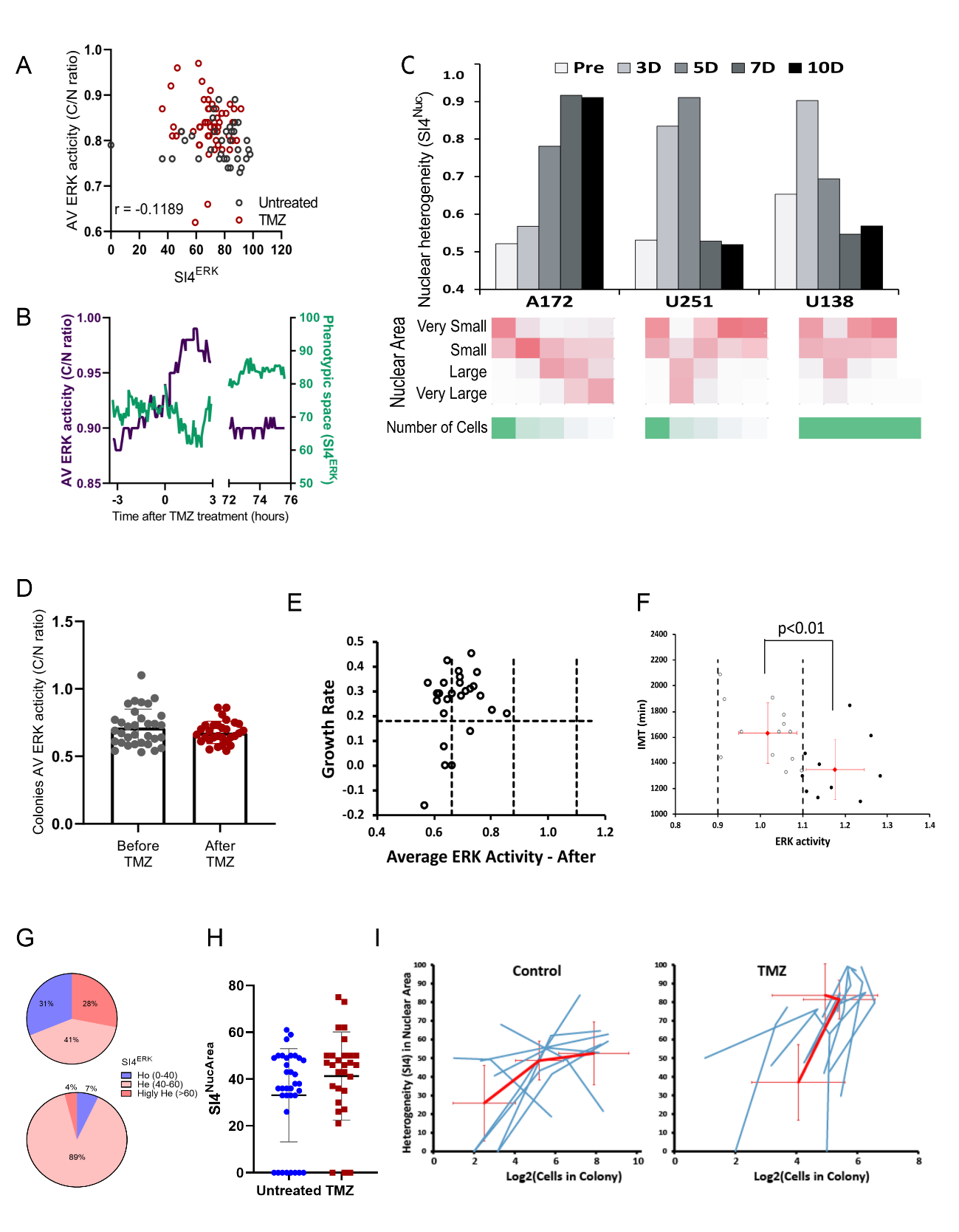
**

**Fig. S3** (A) Correlation between average ERK activity and phenotypic space occupied by untreated and TMZ treated cells. Each dot represents an image field with at least 20 cells. (B) Dynamics of average ERK activity (magenta) and phenotypic space (green) occupied by a population of at least 50 cells prior and during TMZ treatment (100uM for 3h), and 3 days after drug withdraw. Cells were imaged every 10 min for 3 h prior, during TMZ treatment and 3 days after drug removal. (C) Heterogeneity in nuclear area (VS, S, L and VL, respectively: very small, small, large and very large) of 3 glioma cell lines on days 3, 5, 7 and 10 after TMZ treatment withdraw (TMZ 50μM for 3 days). Sensitivity of the cell line to TMZ is shown as a proportion of live cells (green heatmap), while the nuclear area representing cell cycle phase and senescence induction distribution is shown as the proportion of cells in which nuclear area (red heatmap). (D) Average ERK activity of untreated and TMZ treated A172 colonies (100uM for 3h) 3 days after drug removal. Each dot represents the mean ERK activity of cells of a colony imaged every 10 min for 3 h (10x magnification) (n=34 colonies). (E) Colonies of A172 cells categorized according to ERK activity status after TMZ treatment (day 7) in relation to growth rate. TMZ (temozolomide). (F) Average Intermitotic time (IMT) of the two daughter cells in relation to the ERK activity of the mother cell at the last 5h of cell cycle. (G) Proportion of colonies in each ERK phenotypic categories for untreated colonies on day 4 (upper) and TMZ treated colonies after 3 days of drug withdrawal (down). (H) Nuclear area heterogeneity of A172 untreated colonies and TMZ treated colonies (100uM for 3h) 3 days after drug removal. Each dot represents the nuclear area heterogeneity of cells of a colony (10x magnification) (n=34 colonies). (I) Nuclear area heterogeneity of U251 untreated colonies and TMZ treated colonies (50uM for 3 days) on days 0, 3 and 6 after treatment. Blue lines represent the SI4^NucArea^ of a colony along the colony growth. Red lines represent the SI4^NucArea^ average from all colonies (n=12).

**
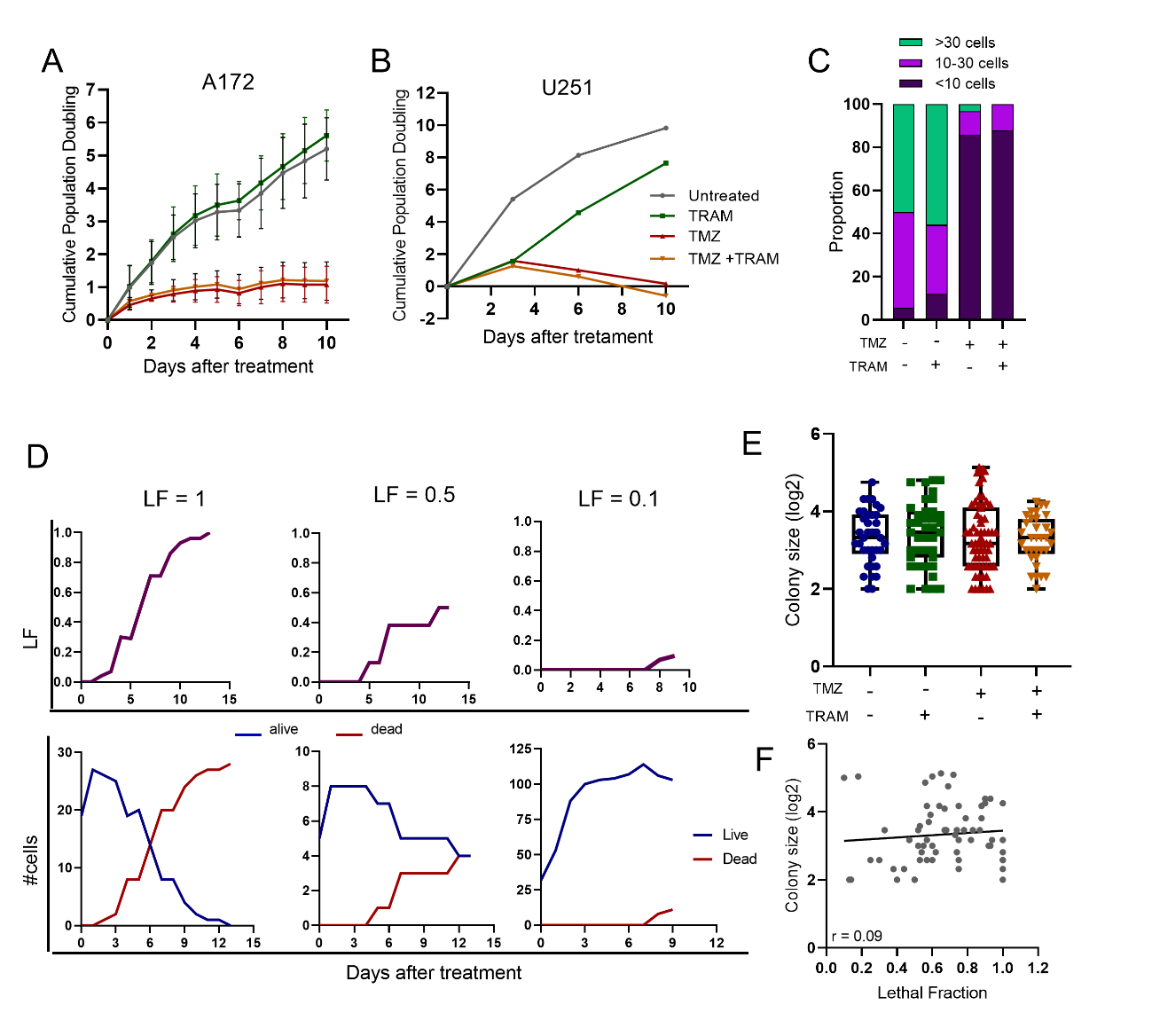
**

**Fig. S4** (A) Cumulative population doubling of A172 and (B) U-251 MG cells treated with TMZ (100uM for 3h), TRAM (20nM for 24h) or a combination of TMZ and TRAM (A172 n=3; U-251 n=1). (C) Proportion of colony sizes of A172 cells at day 14 of growth for untreated (n=34), TMZ (n=64), TRAM (n=25) or TMZ and TRAM (n=33) treated colonies. (D) Representative lethal fraction (LF) scores of A172 cells (above) and corresponding live (blue lines) and dead (red lines) cell counts (below) for colonies with maximum (LF=1), medium (LF=0.5) and minimum (LF=0.1) death rate after TZM treatment. An LF of 0 indicates all cells in the colony remained alive, while an LF of 1 indicates all cells died. (E) Colony size of A172 cells immediately before treatment addition for untreated (n=34), TMZ (n=64), TRAM (n=25) or TMZ and TRAM (n=33) treated colonies. (F) Pearson correlation between colony size (Log2 of number of cells) at the day of treatment (day 4 of growth) and lethal fraction at the end of the experiment (day 14).
